# Health Cure

**Published:** 1886-09

**Authors:** 


### *HEALTH CULTURE*

### AND THE SANITARY WOOLEN SYSTEM,

Is the title of a little hand-book, treating mainly of the prevention of diseases common to this country; but the one thing strenuously advanced is the replacement of the cotton or mixed undergarments, now almost invariably worn, by those of *pure wool*, as a needful sanitary reform. As a guide to health alone the book has great merit. Published by Dr. Jaeger's Sanitary Woolen System Co., 827-9 Broadway, New York City. Price 25 cents.

